# 10-Gingerol Targets Lipid Rafts Associated PI3K/Akt Signaling in Radio-Resistant Triple Negative Breast Cancer Cells

**DOI:** 10.3390/molecules25143164

**Published:** 2020-07-10

**Authors:** Meran Keshawa Ediriweera, Jeong Yong Moon, Yen Thi-Kim Nguyen, Somi Kim Cho

**Affiliations:** 1 Subtropical/tropical organism gene bank, Jeju National University, Jeju 63243, Korea or mk.ediriweera@gmail.com (M.K.E.); owenmjy@jejunu.ac.kr (J.Y.M.); 2 Interdisciplinary Graduate Program in Advanced Convergence Technology and Science, Jeju National University, Jeju 63243, Korea; ntkyen.hcmus@gmail.com; 3 Faculty of Biotechnology, College of Applied Life Sciences, SARI, Jeju National University, Jeju 63243, Korea

**Keywords:** 10-gingerol, ginger, breast cancer, lipid rafts, radio-resistance

## Abstract

10-Gingerol is a major phenolic lipid found in the rhizomes of ginger (*Zingiber officinale*). Being amphiphilic in nature, phenolic lipids have the ability to incorporate into cell membranes and modulate membrane properties. The purpose of the present study was to evaluate the effects of 10-gingerol on lipid raft/membrane raft modulation in radio-resistant triple negative breast cancer (MDA-MB-231/IR) cells. The effects of 10-gingerol on MDA-MB-231/IR cells’ proliferation, clonogenic growth, migration, and invasion were assayed using MTT, colony formation, cell migration, and invasion assays, respectively. Sucrose density gradient centrifugation was used to extract lipid rafts. Western blotting and immunofluorescence were employed to assess the effects of 10-gingerol on lipid raft/membrane raft modulation and lipid rafts-associated PI3K/Akt signaling. Cholesterol measurements were carried out using a commercially available kit. 10-gingerol suppressed the proliferation, migration, invasion, and induced apoptosis through targeting the PI3K/Akt signaling pathway in MDA-MB-231/IR cells. Moreover, 10-gingerol was found to modulate the lipid rafts of MDA-MB-231/IR cells and attenuate the key PI3K/Akt signaling components in lipid rafts. The cholesterol content of the lipid rafts and rafts-resident Akt signaling were also affected by exposure to 10-gingerol. The results of the present study highlight rafts-associated PI3K/Akt signaling as a new target of 10-gingerol in MDA-MB-231/IR cells, thus rationalizing a new rafts-mediated treatment approach for radio-resistant triple negative breast cancer cells.

## 1. Introduction

Breast cancer is the most frequent malignancy in females worldwide and is a molecularly heterogeneous disease [1]. Molecular features associated with tumor heterogeneity play a key role in determining treatment plans for breast cancer [2]. Among the breast cancer subtypes, hormone receptor-positive tumors respond to endocrine therapy, while endocrine therapy is not beneficial for triple negative tumors, as they do not express hormone receptors, making it difficult to treat with currently available breast cancer treatments [2]. Breast cancer patients with metastatic disease are mainly treated with chemo- and radiotherapies to enhance the overall survival. Adverse side effects and development resistance after therapy initiation have been identified as major disadvantages of chemo- and radiotherapies. Therefore, it is of great significance to investigate new anti-breast cancer agents, which can effectively target chemo-and radioresistant breast tumor cells without exerting adverse side effects.

Lipid/membrane rafts are highly dynamic, low-density, cholesterol-rich, sphingolipid-rich domains of the plasma membrane that can serve as platforms for a number of signaling proteins responsible for various molecular and cellular events [3,4]. By compartmentalizing different signaling pathways, lipid rafts have been reported to regulate cellular signaling routes between cell membrane and intracellular sites [3]. Cholesterol plays a key role in lipid rafts organization and stabilization. Notably, the presence of major components of cancer signaling pathways such as PI3K/Akt, EGF/EGFR, RAS, JNK-MMP2/9, and estrogen makes lipid rafts a promising therapeutic target in anti-cancer therapy [3,4]. Cancer cells have been reported to contain higher levels of cholesterol and lipid rafts than their normal counterparts [3,4], and cholesterol has been reported to play a prominent role in the development of radio-resistance [5]. Some studies have demonstrated the role that lipid rafts play in radiotherapy resistance. A recent study has shown that the integrity of lipid rafts remains steady, even after radiation exposure [6]. Another recent study illustrates that radiation can enhance the integrity of lipid rafts in lung cancer cells, which might contribute to the development of radiation resistance [7]. Bionda et al. 2007 have shown that the radio-resistance of head and neck squamous carcinoma is associated with some defects in lipid rafts clustering [8]. Some investigations report that several natural products and synthetic chemicals can modulate the structure and function of lipid rafts [3].

Natural products have been used for a number of clinical applications since ancient times [9]. Ginger is one of the most commonly used condiments in the world. It has a long history of ethnomedicinal use dating back 3000 years [10]. Ginger has been used for the treatment of various ailments, including arthritis, pains, atherosclerosis, depression, common colds, and infections [10]. Among a large variety of phytochemicals, gingerols, paradols, shogaols, and lactones are commonly found in ginger. Additionally, organic extracts of ginger and compounds have been reported to exert numerous pharmacological effects including anti-cancer, anti-oxidant, anti-inflammatory, immunomodulatory, and anti-hyperlipemic properties [10,11].

In the present study, we provide experimental evidence that 10-gingerol, a major phytochemical constituent in ginger, can target lipid raft-associated PI3K/Akt signaling through the modulation of lipid rafts, leading to cell death and apoptosis in radio-resistant triple negative breast cancer cells (MDA-MB-231/IR).

## 2. Results and Discussion

### 2.1. 10-Gingerol Suppresses the Proliferation, Migration, and Invasion of MDA-MB-231/IR Cells

**Figure 1:**
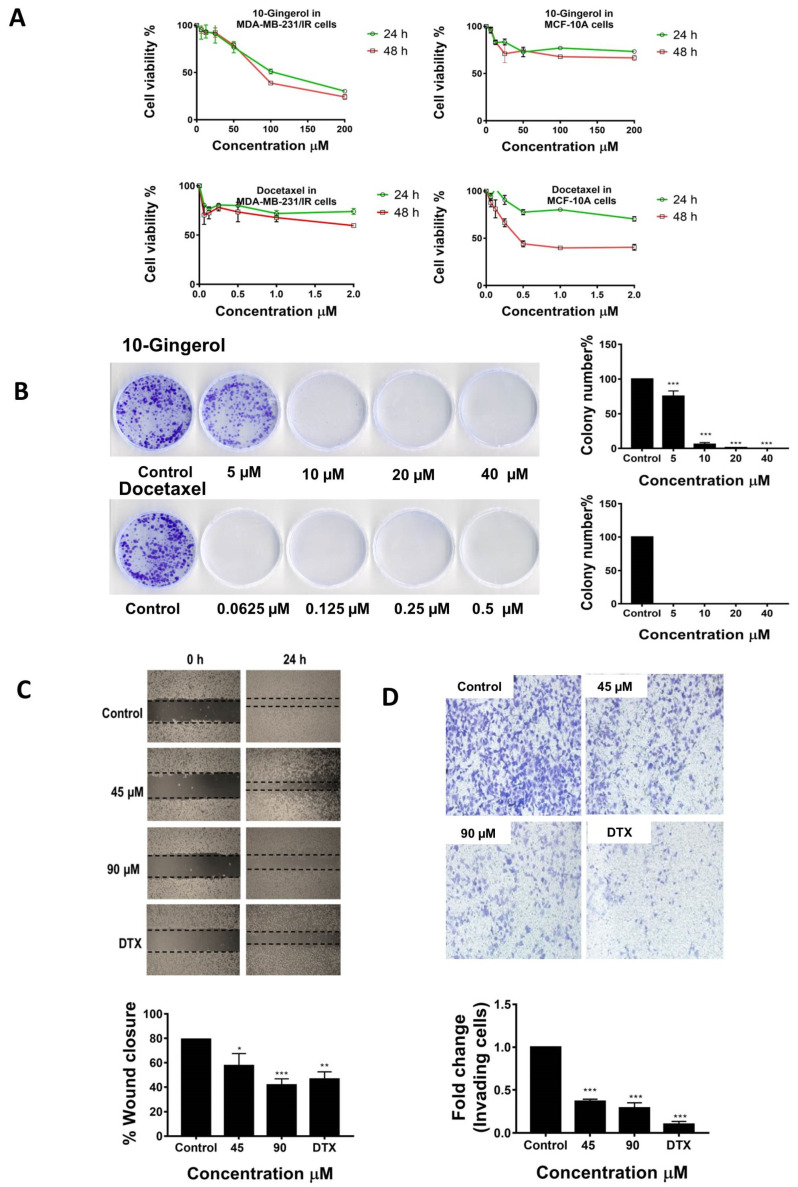
10-gingerol suppresses the proliferation, migration and invasion of MDA-MB-231/IR cells: (**A**) Anti-proliferative effects of 10-gingerol and docetaxel (DTX) in MDA-MB-231/IR and MCF-10A cells. (**B**) Effects of 10-gingerol and docetaxel on the clonogenic ability of MDA-MB-231/IR cells. (**C** and **D**) 10-gingerol significantly inhibits migration and invasion of MDA-MB-231/IR cells. DTX-3 µM of docetaxel. * *p* < 0.05, ** *p* < 0.01, and *** *p* < 0.001 compared with untreated cells.

According to our recent studies, the established MDA-MB-231/IR cells were found to possess enhanced chemo-and radio-resistance and several differentially regulated genes (DEGs) related to cell division, metabolism, drug resistance, metastasis, and stem-like properties, compared with parental MDA-MB-231 cells [12]. To assess the inhibitory effects of 10-gingerol on the proliferation of MDA-MB-231/IR cells, cells were exposed to different concentrations (200, 100, 50, 25, 12.5, and 6.25 µM) of 10-gingerol and incubated for 24 and 48 h. Docetaxel, a clinically used anti-cancer drug (an anti-mitotic agent), was used as the positive control. The MTT assay results demonstrated that 10-gingerol can suppress the proliferation of MDA-MB-231/IR cells in a dose and time-dependent manner (Figure 1A). The doses of 10-gingerol causing 50% growth inhibition (IC_50_) of MDA-MB-231/IR cells at 24 and 48 h incubations were 121.2 and 101.4 µM, respectively. In contrast, 10-gingerol demonstrated fewer anti-proliferative effects in parental MDA-MB-231 cells (Figure S1 of Supplementary Materials 1), indicating a selective anti-proliferative pattern toward radio-resistant MDA-MB-231 cells. In addition, 10-gingerol exerted fewer cytotoxic effects in MCF-10A normal mammary epithelial cells than the positive control docetaxel at two post-incubation periods [IC_50_ 10-gingerol: 327.8 µM (24 h), and 219.8 µM (48 h); docetaxel: 3.03 µM (24 h) and 1.78 µM (48 h)] (Figure 1A).

The anti-proliferative effects of 10-gingerol were further confirmed by the colony formation assay. As shown in Figure 1B, the exposure of 10-gingerol significantly reduced the clonogenic ability of MDA-MB-231/IR cells. Docetaxel also demonstrated anti-clonogenic effects in MDA-MB-231/IR cells (Figure 1B). To investigate the effects of 10-gingerol on MDA-MB-231/IR cell migration, wound-healing assay was conducted. The migratory ability of MDA-MB-231/IR cells was significantly affected by 10-gingerol exposure (Figure 1C). A similar inhibitory pattern was also evident in the docetaxel-treated MDA-MB-231/IR cells (Figure 1C). Next, the impact of 10-gingerol on MDA-MB-231/IR cell invasion was assessed using the Transwell cell invasion assay. The results indicated that 10-gingerol can significantly reduce the number of invading MDA-MB-231/IR cells (Figure 1D). The positive control docetaxel also exerted inhibitory effects on MDA-MB-231/IR cell invasion (Figure 1D). Overall, the results highlight the ability of 10-gingerol to target MDA-MB-231/IR cell proliferation, colony formation, migration, and invasion, while mediating less cytotoxicity to normal mammary epithelial cells.

### 2.2. 10-Gingerol Induces Apoptosis and Targets PI3K/Akt Signaling in MDA-MB-231/IR Cells

**Figure 2:**
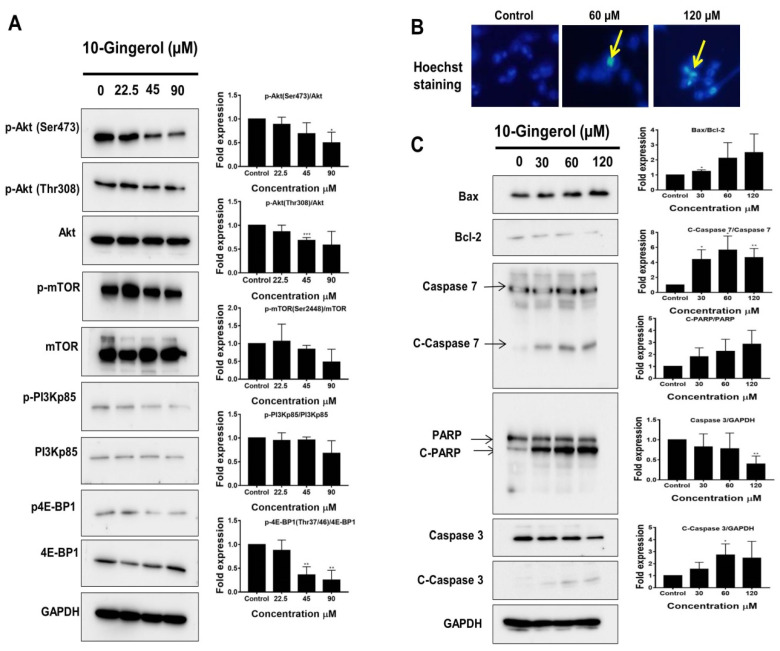
10-gingerol suppresses the expression of the PI3K/Akt signaling pathway components and induces apoptosis in MDA-MB-231/IR cells: (**A**) Effects of 10-gingerol on the expression of PI3K/Akt signaling pathway component. Representative bands for p-Akt (Ser473), p-Akt (Thr308), Akt, p-mTOR, mTOR, p-PI3Kp85, PI3Kp85, p4E-BP1, and 4E-BP1 proteins. (**B** and **C**) 10-gingerol induces apoptosis in MDA-MB-231/IR cells. Apoptosis in MDA-MB-231/IR cells was confirmed by Hoechst staining (**B**) and Western blot experiments (**C**). In Figure 2B, yellow arrows indicate condensed chromatin. GAPDH was used as the internal control. * *p* < 0.05, ** *p* < 0.01 and *** *p* < 0.001 compared with untreated cells.

Abnormal regulation of the PI3K/Akt/mTOR signaling pathway is very common in most of the human cancers, making this pathway as an important pharmacological target in anti-cancer treatments [13]. To assess the effects of 10-gingerol on PI3K/Akt signaling in MDA-MB-231/IR cells, the expression levels of PI3K/Akt/mTOR signaling components, namely p-Akt (Ser473), p-Akt (Thr308), p-mTOR (Ser2448), p-PI3Kp85, and p4E-BP1 were evaluated using Western blot. A dose-dependent decrease in the phosphorylation of p-Akt (Ser473), p-Akt (Thr308), p-PI3Kp85, and p4E-BP1 was observed in MDA-MB-231/IR cells exposed to 10-gingerol for 24 h (Figure 2A). Notably, the decreased phosphorylation of p-mTOR (Ser2448) was only observed at the last two doses of 10-gingerol (Figure 2A).

Evading programmed cell death (apoptosis) is a key feature of cancer cells [14]. Therefore, apoptosis promoting agents in cancer cells are considered as key candidates in anti-cancer treatments. A number of natural compounds including ginger-derived compounds have been reported to induce apoptosis in a range of human cancer cells [10]. To explore whether the cytotoxicity of 10-gingerol is mediated through the induction of apoptosis in MDA-MB-231/IR cells, Hoechst staining was first carried out. Following treatment with 10-gingerol, chromatin condensation was visible in MDA-MB-231/IR cells (Figure 2B). In addition, the expression of Bax and Bcl-2, cleaved caspase-7, cleaved PARP, caspase 3, and cleaved caspase 3 following 10-gingerol exposure was analyzed by Western blot experiments. As shown in Figure 2B, 10-gingerol activated the expression of some markers of apoptosis including Bax/Bcl-2, cleaved caspase-7, cleaved PARP, and cleaved caspase-3 compared with untreated cells. Collectively, these results indicate that 10-gingerol can induce apoptosis in MDA-MB-231/IR cells through inhibition of the PI3K/Akt/mTOR signaling pathway.

Similar to this observation, several studies have demonstrated the ability of ginger compounds (gingerols, paradols, and shogaols) to induce apoptosis in a variety of cancer cells in vitro and in vivo [10,11]. In a recent investigation, 10-gingerol has been reported to target MDA-MB-231 cell metastasis [15]. A study by Zhang et al. 2017 demonstrated that 10-gingerol can induce apoptosis by targeting PI3K/Akt signaling in HeLa cells [16]. Another investigation demonstrates that 10-gingerol can suppress the growth of ovarian cancer cells by inducing cell cycle arrest [17]. 6-gingerol, a structurally similar compound to 10-gingerol, has been reported to sensitize gastric cancer cells to cisplatin through the inhibition of PI3K/Akt signaling [18].

### 2.3. Lipid Raft Modulation by 10-Gingerol Results in Displacement of Raft-Located PI3K/Akt Signaling Components

Phenolic lipids or resorcinolic lipids are amphiphilic in nature due to the presence of hydrophobic alkyl side chains attached to the hydrophilic dihydroxybenzene ring [19,20]. Having hydrophobic properties, phenolic lipids can easily incorporate into cell membranes and cause significant alterations in the membrane environment and properties [19]. Some phenolic lipids have been reported to strongly interact with phospholipid bilayers and alter the functions of membrane proteins [19,21]. These reports, which describe the ability of phenolic lipids to interact with biological membranes and alter the functions of membrane-associated proteins, provided a rational to investigate a possible similar role of 10-gingerol, a ginger-derived phenolic lipid [21], in membrane-related activities. By considering the structure of 10-gingerol, the composition of lipid rafts, and previous reports describing the effects of phenolic lipids on altering membrane properties, we hypothesized that 10-gingerol can modulate MDA-MB-231/IR membrane rafts.

**Figure 3:**
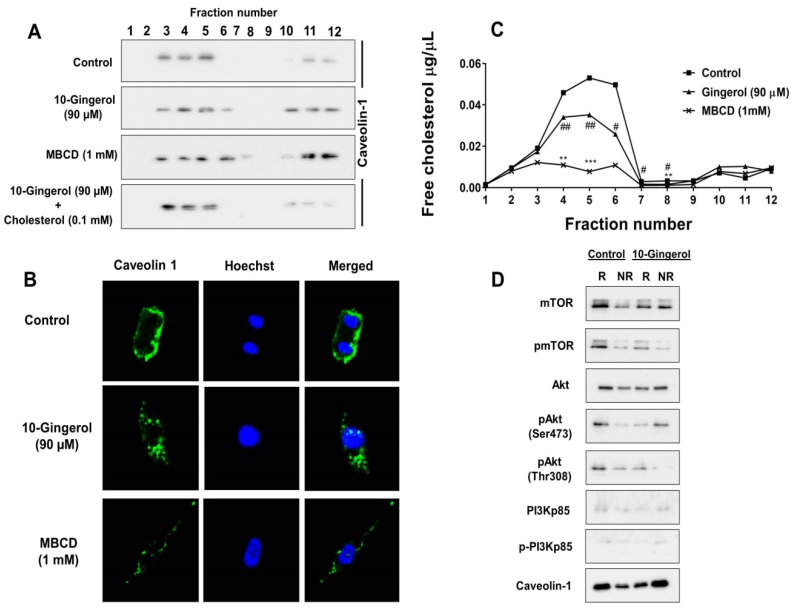
10-gingerol modulates lipid rafts of MDA-MB-231/IR cells and displaces rafts-resident PI3K/Akt signaling components: (**A**) Distribution of caveolin-1 in lipid rafts (3–5) and non-raft fractions (10–12) of MDA-MB-231/IR cells. Following serum starvation, cells were treated with 10-gingerol (90 µM), 10-gingerol (90 µM), plus 0.1 mM cholesterol or methyl-β-cyclodextrin (MBCD) (1 mM) for 24 h, and lipid rafts were isolated as described in methods. Following sucrose-density gradient centrifugation, equal volumes (20 µL) of collected fractions were subjected to Western blot and analyzed using lipid raft marker caveolin-1. (**B**) The presence/distribution of lipid rafts marker caveolin-1 in cell membranes was examined in 10-gingerol (90 µM) or MBCD (1 mM) treated MDA-MB-231/IR cells using immunofluorescence. (**C**) Estimation of lipid raft cholesterol levels in 10-gingerol (90 µM) or MBCD (1 mM) treated MDA-MB-231/IR cells cultured in serum-starved DMEM. (**D**) Displacement of rafts-resident PI3K/Akt signaling components following 10-gingerol exposure. Lipid raft fractions were isolated from serum-starved MDA-MB-231/IR cells and, fractions 3–5 (rafts) and 10–12 (non-rafts) were pooled. Proteins were precipitated from 1 mL of pooled sets using methanol and chloroform [31]. The resulted protein pellets were dissolved in equal volumes (50 µL) of loading buffer and subjected to Western blot analysis using the indicated antibodies. * or # *p* < 0.05, ** or ## *p* < 0.01 and *** *p* < 0.001 compared with untreated cells. R: raft fractions and NR: non-raft fractions.

By employing the SwissADME web server, the lipophilicity of 10-gingerol was first predicted. Similar to other phenolic lipids, 10-gingerol was found to have high octanol–water partition coefficients, indicating strong hydrophobic interactions with cell membranes (Figure S2 of Supplementary Materials 1). Then, we investigated the distribution of caveolin-1, a marker for lipid rafts, in MDA-MB-231/IR cell lipid rafts after 10-gingerol exposure (Figure 3A). MDA-MB-231/IR cell lipid rafts (fractions 3–5) possess higher levels of caveolin-1 compared with non-raft fractions (fractions 10–12) (Figure 3A). Compared with the untreated control, 10-gingerol exposure resulted in the shifting of caveolin-1 from lipid raft fractions to non-raft fractions (Figure 3A), which indicated a possible lipid rafts modulatory ability/role of 10-gingerol. Methyl-β-cyclodextrin (MBCD), a raft-disrupting agent by depleting cholesterol [22], was used as the positive control. Importantly, it is worth stating that 10-gingerol does not decrease the total expression of caveolin-1 in MDA-MB-231/IR cells (Figure S3 of Supplementary Materials 1). Interestingly, 10-gingerol and cholesterol co-treatment recovered caveolin-1 in lipid rafts fractions (Figure 3A), suggesting that 10-gingerol might influence the cholesterol content of MDA-MB-231/IR cell lipid rafts. An analysis of distribution of lipid raft markers caveolin-1 or 2 in raft and non-raft fractions has been used in experiments assessing lipid raft disruption in studies [22,23]. Furthermore, several natural compounds such as γ-Tocotrienol [24], betulinic acid [25], anandamide [26], epigallocatechin-3-gallate [27], resveratrol [22], n-3 polyunsaturated fatty acids [28], and curcumin [29] have been shown to disrupt/modulate lipid rafts.

The modulation of lipid rafts by 10-gingerol was further confirmed by immunofluorescence. As shown in Figure 3B, the localization of caveolin-1 in the membrane was greatly affected by 10-gingerol and MBCD treatments after 24 h of incubation. As 10-gingerol and cholesterol co-treatment recovered caveolin-1 in lipid raft fractions, total cholesterol levels were estimated in lipid raft fractions of MDA-MB-231/IR cells exposed to 10-gingerol and MBCD. Lipid rafts fractions of MDA-MB-231/IR cells were found to contain higher levels of total cholesterol than the non-raft fractions (Figure 3C). As we assumed that 10-gingerol might influence the cholesterol content of lipid rafts, 10-gingerol treatment caused a reduction in total cholesterol levels in MDA-MB-231/IR cell lipid raft fractions (Figure 3C). A dramatic reduction in total cholesterol levels was observed in MBCD-treated cells (Figure 3C). Notably, according to the lipid raft cholesterol analysis of MDA-MB-231 and MDA-MB-231/IR cells, MDA-MB-231/IR cells were found to contain significantly higher amounts of raft-associated cholesterol, which might indicate an increased affinity of 10-gingerol toward cholesterol-rich lipid rafts of MDA-MB-231/IR cells (Figure S4 of Supplementary Materials 1). Similar to our findings, studies have also reported high cholesterol levels in lipid raft fractions compared with non-raft fractions [30].

The PI3K/Akt signaling pathway is frequently dysregulated in a range of human cancers, including breast cancer [13]. The main components of the PI3K/Akt signaling pathway have been reported to associate with membrane rafts [4]. As the PI3K/Akt signaling pathway is a main target of 10-gingerol in MDA-MB-231/IR cells (Figure 2A) and 10-gingerol was found to modulate lipid rafts in MDA-MB-231/IR cells (Figure 3A,B), we analyzed the occurrence of main PI3K/Akt signaling components in MDA-MB-231/IR cell lipid rafts following 10-gingerol exposure. As shown in Figure 3D, the major components of the PI3K/Akt signaling pathway, including activated forms of Akt, mTOR, and PI3Kp85, are abundant in the lipid rafts of MDA-MB-231/IR cells, indicating that membrane rafts can serve as a key platform for PI3K/Akt signaling components in radio-resistant MDA-MB-231 cells. Following 10-gingerol exposure, displacement of major components of the PI3K/Akt signaling pathway in the lipid rafts was evident (Figure 3D), suggesting that 10-gingerol can displace PI3K/Akt signaling pathway components through the modulation/disruption of lipid rafts in MDA-MB-231/IR cells. Caveolin-1 was used as the lipid rafts marker (Figure 3D).

### 2.4. 10-Gingerol Affects Rafts-Resident Akt Signaling and Akt Downstream Targets

**Figure 4:**
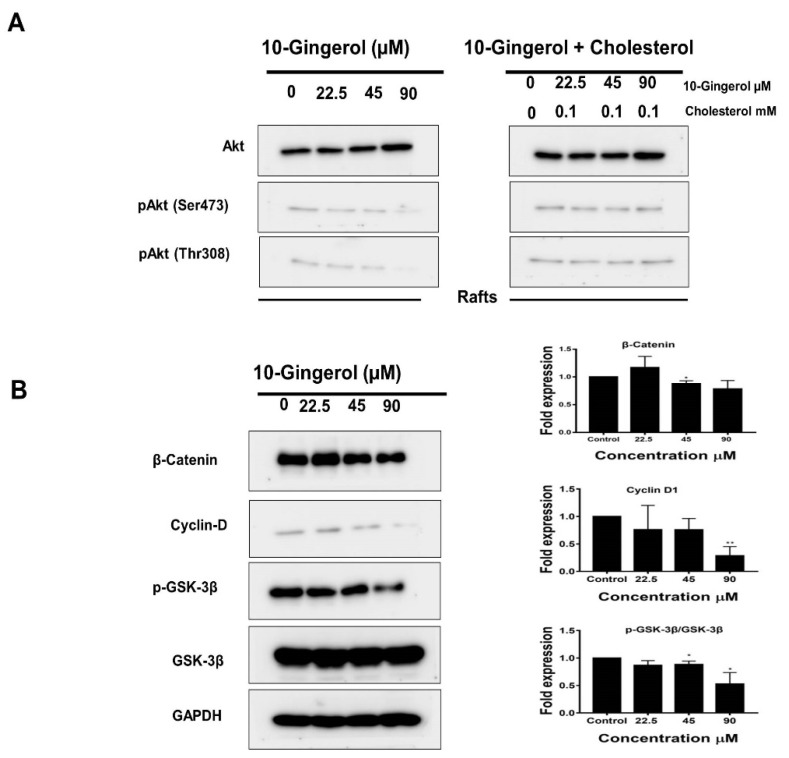
Effects of 10-gingerol on Akt signaling in lipid rafts and Akt downstream targets: (**A**) After isolating lipid rafts fractions, fractions 3–5 were pooled, and proteins were precipitated. Following protein normalization, equal amounts of proteins were subjected to Western blot and analyzed using indicated antibodies. 10-gingerol exposure resulted in a dramatic reduction of rafts-resident p-Akt (Ser473) and p-Akt (Thr308) levels. The co-treatment of cholesterol (0.1 mM) and 10-gingerol (90 µM) appeared to increase p-Akt(Ser473) and p-Akt(Thr308) levels in lipid rafts. (**B**) 10-gingerol suppressed the expression of Akt downstream target proteins. GAPDH was used as the internal control. * *p* < 0.05 and ** *p* < 0.01 compared with untreated cells.

Upon activation, phosphatidylinositol (3,4,5)-trisphosphate (PIP3) recruits Akt to the plasma membrane [13]. The integrity of lipid rafts and raft-associated cholesterol has been reported to be responsible for the activation of Akt in lipid rafts [32]. By employing Western blot experiments, we investigated whether 10-gingerol could alter the expression of activated Akt in lipid rafts. As shown in Figure 4A, 10-gingerol was found to dephosphorylate pAkt (Ser473) and pAkt (Thr308) in lipid rafts dose-dependently. The co-treatment of 10-gingerol and cholesterol almost restored the phosphorylation status of pAkt (Ser473) and pAkt (Thr308) in lipid rafts (Figure 4A), suggesting a possible mechanism by which 10-gingerol reduces the integrity of lipid rafts through the depletion of raft cholesterol. However, detailed studies are necessary to elucidate how 10-gingerol depletes raft-resident cholesterol in MDA-MB-231/IR cells.

Akt promotes the cell cycle through the phosphorylation of GSK3β at Ser9, and this phosphorylation inactivates GSK3β kinase [33]. Inactive GSK3β has been reported to stabilize the expression of β-catenin and up-regulate cyclin D1, thereby promoting breast cancer tumorigenesis [34]. We next investigated whether 10-gingerol can alter the expression of Akt downstream targets in MDA-MB-231/IR cells. As shown in Figure 4B, 10-gingerol down-regulated Akt downstream targets GSK3β and cyclin D1 in a dose-dependent manner. β-catenin down-regulation was only observed at the last two doses of 10-gingerol (Figure 4B). Studies have also demonstrated the effects of ginger constituents on the expression of Akt downstream targets in lung and cervical cancer cells [35,36].

## 3. Materials and Methods

### 3.1. Cell Lines, Chemicals, Reagents, Antibodies, and Kits

A radio-resistant cell line (MDA-MB-231/IR) was recently developed in our laboratory [12]. Cell culture media [Dulbecco’s modified Eagle’s medium (DMEM)], fetal bovine serum (FBS), and antibiotics were purchased from Invitrogen (Carlsbad, CA, USA). 10-gingerol (Item number: 11842; ≥98% purity) was purchased from Cayman chemicals (Ann Arbor, MI, USA). All other chemicals used in experiments were purchased from Sigma Aldrich Chemical Co. (St. Louis, MO, USA) unless otherwise indicated. Primary antibodies caspase-3, cleaved-caspase-3, caspase-7, Bax, Bcl-2, PARP, cyclin D1, GAPDH, PI3 Kinase p85, p-PI3 Kinase p85α, Akt, p-Akt (Ser473), p-Akt (Thr308), mTOR, p-mTOR, 4E-BP1, p-4E-BP1, caveolin-1, GSK-3β, and p-GSK-3β were purchased from Cell Signaling Technology (Beverly, MA, USA). β-catenin was purchased from BD bio-sciences (San Diego, CA, USA). HRP-conjugated secondary antibodies (goat anti-rabbit and anti-mouse IgG) were purchased from Vector Laboratories (Burlingame, CA, USA). An ECL Plus kit (western blotting substrate) was purchased from Biosesang Inc. (Seongnam, Korea).

### 3.2. Cell Culture

The MDA-MB-231 (ATCC: HTB-26) and MDA-MB-231/IR cells were cultured in DMEM supplemented with FBS (10%) and antibiotics (100 U/mL streptomycin and 100 U/mL penicillin). MCF-10A cells (ATCC: CRL-10317) were cultured in mammary epithelial cell growth medium (Lonza, Walkersville, MD, USA) supplemented with recommend growth factors (insulin, hydrocortisone, epidermal growth factor, and bovine pituitary extract). Cells were incubated at 37 °C in a 5% CO2 incubator and harvested by trypsinization following 80% confluency.

### 3.3. Cell Proliferation Assay

The MTT assay was used as the cell proliferation assay in the present investigation. MDA-MB-231, MDA-MB-231/IR, and MCF-10A cells were cultured in 96-well plates (5000 cells/well) and incubated for 24 h. Following incubation, cells were exposed to 10-gingerol (200, 100, 50, 25, 12.5, and 6.25 µM) for 24 and 48 h. MTT assay was conducted as described in our recent publication [12]. The percentage of cell viabilities of treated and untreated groups were calculated using the formula (control − treated) ÷ control) × 100%. GraphPad Prism 5 software was used to calculate IC_50_ values. Briefly, following 24 h exposure with 10-gingerol, cells were washed with PBS (phosphate-buffered saline) and MTT solution (0.5 mg/mL) dissolved in PBS was added to each well and incubated for 4 h at 37 °C. Following incubation, 100 µL of dimethyl sulfoxide (DMSO) was added each well. A microplate reader was used to measure the absorbance at 570 nm.

### 3.4. Colony Formation Assay

The MDA-MB-231/IR cells (200 cells/dish) were seeded in cell culture dishes (60 mm) and incubated for 24 h. Following 24 h incubation, cells were treated with 10-gingerol (40, 20, 10, and 5 µM) and incubated for 10 days. After 10 days incubation, cell colonies were fixed and stained with crystal violet. Docetaxel (0.5, 0.25, 0.125, and 0.0625 µM) was used as the positive control. Colonies were manually counted and expressed as percentage of control.

### 3.5. Wound Healing Assay

The MDA-MB-231/IR cells (1 × 10^5^) were seeded in 6-well plates and allowed to reach 95% confluence. Upon 95% confluency, the cell monolayer was scratched with a sterile pipette tip. Then, cells were washed with PBS and exposed to 10-gingerol (90 and 45 µM) for 24 h. Following incubation, the gap area was quantified and compared with controls (0 h). Docetaxel (3 µM) was used as the positive control in this experiment.

### 3.6. Trans-Well Cell Invasion Assay

Trans-well cell invasion assay (Corning, Cambridge, MA, USA) was conducted as previously described by us [12]. After coating the upper chamber with 1% matrigel, MDA-MB-231/IR cells (1 × 10^5^ cells/trans well) were transferred to the upper chamber (supplemented with or free of, 10-gingerol). The lower chamber received DMEM containing 10% FBS. After 24 h post-incubation, cells were fixed and stained with 2% crystal violet. Cells were photographed under a phase-contrast microscope. After calculating invaded cell densities in control and treated groups using the image J software, cell densities in treated groups were normalized to control groups to obtain fold changes.

### 3.7. Western Blot Experiments

Following treatments, cell lysates were prepared using radioimmunoprecipitation buffer (RIPA). After estimating protein concentrations (using Pierce™ BCA Protein Assay Kit, Thermo Fisher Scientific), equal amounts of proteins were subjected to SDS-PAGE and transferred to PVDF membranes electrophoretically at 150 V for 1 h. After blocking with skim milk (5%), membranes were exposed to primary antibodies at 4 °C overnight. All the primary antibodies were diluted in skim milk (1:1000). After overnight incubation, membranes were washed three times with tris-buffered saline and incubated with suitable secondary antibodies (1:5000) for 1 h at room temperature. After washing three times with TBST, an ECL Plus Kit was used to develop bands, and developed bands were observed using the ChemiDoc touch imaging system (Bio-Rad Laboratories, Hercules, CA, USA).

### 3.8. Isolation of Lipid Rafts

Lipid rafts were isolated using a previously described method with modifications [22]. Serum-starved MDA-MB-231/IR cells (1 × 10^7^) were washed with PBS, lysed in 1.5 mL of lysis buffer (0.5% Triton X-100, 15 mM Tris, 1 mM EDTA, 100 mM NaCl, 50 mM phenylmethylsulfonyl fluoride (PMSF), 5% glycerol and anti-proteases), and homogenized for 30 min on ice. The resulting cell lysate (1 mL) was mixed with 1 mL of lysis buffer comprising 80% sucrose and carefully transferred to 7.5 mL of lysis buffer containing 30% sucrose followed by 3 mL of buffer comprising 5% sucrose (layering of sucrose) and centrifuged at 20,000 rpm for 18 h prior to collecting 12 1 mL fractions. Fractions 3–5 contain lipid rafts, while fractions 10–12 are considered non-raft fractions [37].

### 3.9. Immunofluorescence and Imaging

Serum-starved MDA-MB-231/IR cells (5 × 10^4^) attached to coverslips were fixed with 4% paraformaldehyde for 15 min at room temperature. Following fixing, cells were washed three times with PBS and blocked with 1% bovine serum albumin (BSA). Then, cells were incubated with caveolin-1 primary antibody overnight at 4 °C, washed with PBS three times, and incubated with Alexa 488-conjugated secondary antibody for 1 h at room temperature. After incubation, cover slips were washed with PBS, and cells were counter stained with Hoechst 33342 (5 µg/mL). Coverslips were observed using a digital cell imaging system (CELENA-S, Logos Bio-systems, Korea).

### 3.10. Measurement of Cholesterol Levels

An EZ-total cholesterol assay kit (DoGen Bio Co., Ltd., Seoul, Korea) was used for cholesterol measurements according to the manufacturer’s instructions.

### 3.11. Prediction of Lipophilicity of 10-gingerol

The lipophilicity of 10-gingerol was predicted with the help of the SwissADME tool, which is a free online tool available for absorption, distribution, metabolism, excretion, and toxicity (ADME) predictions [38].

### 3.12. Statistical Analysis

ImageJ software (US National Institutes of Health, Bethesda, MD, USA) was utilized for densitometry measurements of blots. Statistical analysis was performed using the GraphPad Prism 5 (GraphPad Software, Inc., La Jolla, CA, USA). Data in the present study are presented as the mean ± standard deviation of three separate experiments. For group comparisons, one way analysis of variance (ANOVA) with Dunnett test and student t-test were used at *p* < 0.05, *p* < 0.01, or *p* < 0.001.

## 4. Conclusions

In conclusion, our investigation found that 10-gingerol, a phenolic lipid found in ginger, can suppress proliferation, migration, and invasion and induce apoptosis in radio-resistant triple negative breast cancer cells (MDA-MB-231/IR) via targeting the PI3K/Akt signaling pathway. Similar to other amphiphilic phenolic lipids, 10-gingerol was found to modulate the cell membrane environment, which was assessed by analyzing MDA-MB-231/IR cells lipid rafts/membrane rafts, lipid raft cholesterol levels, and raft-associated PI3K/Akt signaling components. The overall findings highlight raft-resident PI3K/Akt signaling as a key biological target of 10-gingerol in radio-resistant breast cancer cells.

## Supplementary Materials

The following are available online at https://www.mdpi.com/1420-3049/25/14/3164/s1, Figure S1: Effects of 10-gingerol on MDA-MB-231 cell viability, Figure S2: Lipophilicity prediction of 10-gingerol using the SwissADME webserver, Figure S3: Effects of 10-gingerol on the total expression of caveolin-1 in MDA-MB-231/IR cells, Figure S4: Comparison of lipid rafts cholesterol levels in MDA-MB-231 and MDA-MB-231/IR cells.
