# Supplementary material for: 10-Gingerol Targets Lipid Rafts Associated PI3K/Akt Signaling in Radio-Resistant Triple Negative Breast Cancer Cells
Source: Molecules. 2020 Jul 10;25(14):3164. doi: 10.3390/molecules25143164 (PMC7397170; doi:10.3390/molecules25143164)

10-Gingerol targets lipid rafts associated PI3K/Akt signaling in radio-resistant triple negative breast cancer cells.

Supplementary file 1

Figure S1: Effects of 10-gingerol on MDA-MB-231 cell viability

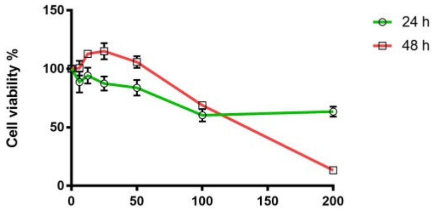

IC_{50}- 234.4 µM (24 h) and 191.9 µM (48 h)

Figure S2: Lipophilicity prediction of 10-gingerol using the SwissADME webserver

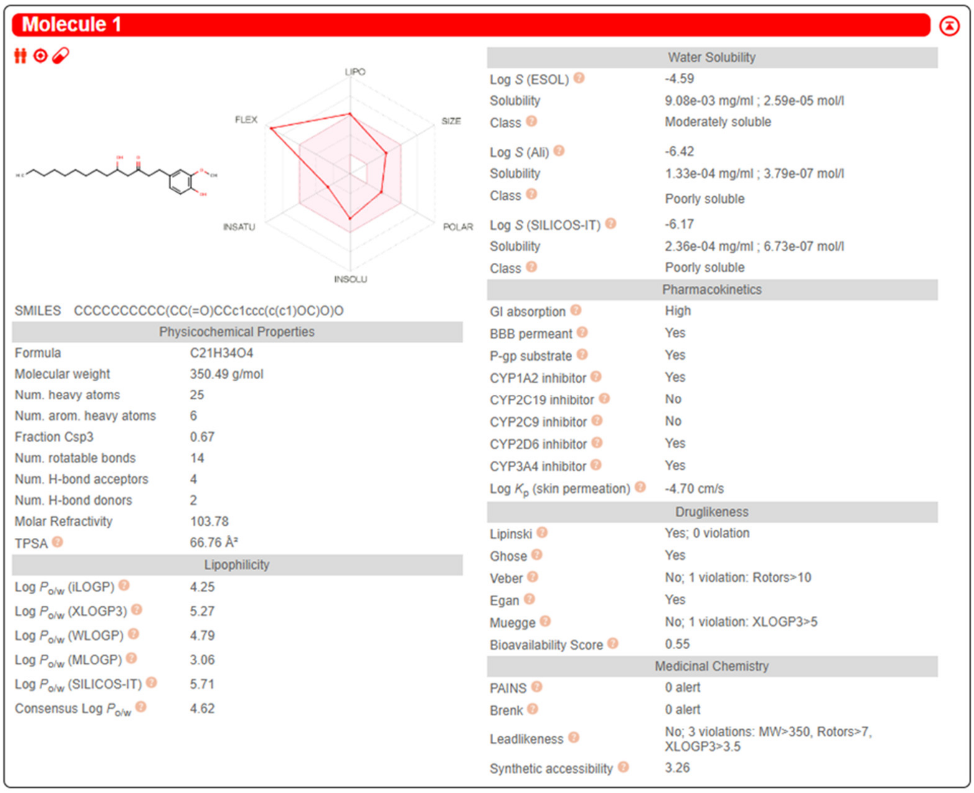

Figure S3: Effects of 10-gingerol on the total expression of caveolin-1 in MDA-MB-231/IR cells

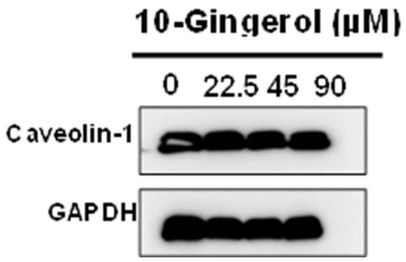

Figure S4. Comparison of lipid rafts cholesterol levels in MDA-MB-231 and MDA-MB-231/IR cells. *P<0.05 and ***P<0.001 compared with MDA-MB-231 cells.

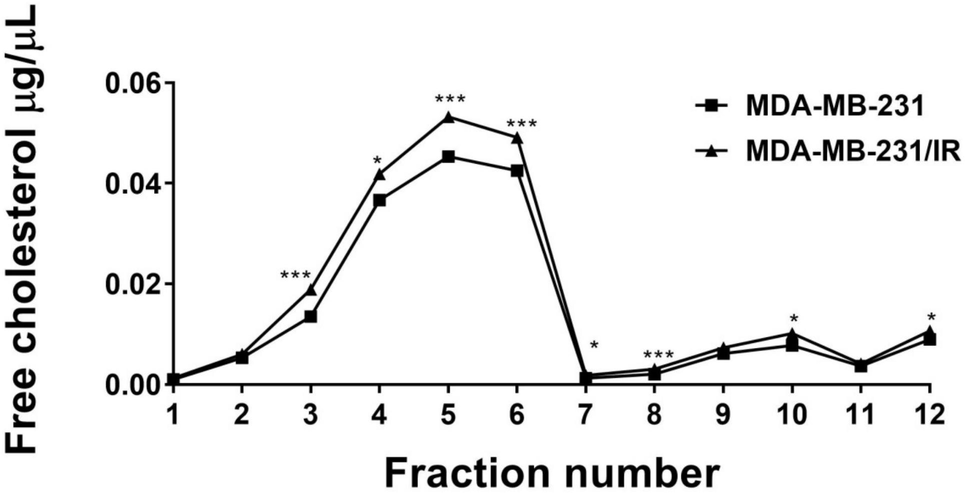

Supplement: Supplementary file 1 [file molecules-25-03164-s001.pdf]
